# Sibling Relations in Patchwork Families: Co-residence Is More Influential Than Genetic Relatedness

**DOI:** 10.3389/fpsyg.2020.00993

**Published:** 2020-06-09

**Authors:** Petra Gyuris, Luca Kozma, Zsolt Kisander, András Láng, Tas Ferencz, Ferenc Kocsor

**Affiliations:** ^1^Institute of Psychology, Faculty of Humanities, University of Pécs, Pécs, Hungary; ^2^Institute of Behavioural Sciences, Medical School, University of Pécs, Pécs, Hungary

**Keywords:** siblings, family relations, parent–offspring conflict, inclusive fitness, cooperation

## Abstract

In “patchwork” families, full siblings, maternal and paternal half-siblings, and non-related children are raised together, and sometimes, genetically related children are separated. As their number is steadily growing, the investigation of the factors that influence within-family relations is becoming more important. Our aim was to explore whether people differentiate between half- and full-siblings in their social relations as implied by the theory of inclusive fitness, and to test whether co-residence or genetic relatedness improves sibling relations to a larger extent. We administered the Sibling Relationship Questionnaire to 196 individuals who were in contact with full-, half-, or step-siblings in their childhood. We built Generalized Linear Mixed Models models to test for the effects of relatedness and co-residence on sibling relations. In general, a higher degree of relatedness was associated with better sibling relations, but only among those who did not live together during childhood. Co-resident siblings’ overall pattern of relation quality was not influenced by the actual level of genetic relatedness. In contrast to this, full siblings reported having experienced more conflicts during childhood than half-siblings, possibly resulting from enhanced competition for the same parental resources. The results suggest that inclusive fitness drives siblings’ relations even in recent industrial societies. However, among individuals who live together, the effect of relatedness might be obscured by fitness interdependence and the subjective feeling of kinship.

## Introduction

### Serial Monogamy and Patchwork Families

Theories on the mating pattern of prehistoric *Homo sapiens* and its hominid predecessors (Miller, 2011; Fisher, 2016) suggest that exclusive, monogamous relationships lasted only for a couple of years, typically until the weaning of the offspring. After this period pairs split up and formed new relationships. In this mating pattern, also called *serial monogamy*, the costs of raising a child were mostly charged on the mother, and children usually stayed with her until reaching full independence. Examples from extant hunter–gatherer societies are consonant with this theory. For instance, at the African Aka people, 18% of children between 11 and 15 years live together with their biological mother and a stepfather (Hewlett, 1993). This figure for the Peruvian Yora tribe is 12.5% (Sugiyama and Chacon, 2005). Taking the reproductively active period of recent hunter–gatherers into account (Blurton Jones, 1987), females, on average, give birth to four to five children in 4- to 5-year intervals from sequential monogamous relations. As a result, each individual might have three to four maternal half-siblings, and even more paternal half-siblings, with an age range of more than 20 years. The occurrence of serial monogamy in industrialized Western societies is similar to what has been observed in preindustrial societies. Some calculations show that 30% of American children live with a stepparent, typically with a stepfather (Bumpass et al., 1995; Anderson, 2011). This rate in Europe varies between 1.1% (Italy) and 11.4% (Czech Republic) (Heuveline et al., 2003). On average, children born in the United States spend 1.9 years in their first 15 years in a patchwork family where a stepfather is present. In Hungary, this number is 1 year (Murinko and Földházi, 2012). While in polygamous societies paternal half-siblings are those who typically live in the same household, in Western monogamous societies, the co-residence of maternal half-siblings is more prevalent (De Graaf, 1997, cited in Pollet, 2007; Tanskanen and Danielsbacka, 2014).

Serial monogamy was likely typical throughout human evolutionary history and still is. Consequently, living in patchwork families, which are complex networks of differentially related individuals, brought recurrent adaptive problems of detecting kinship, allocating resources, supporting those in need, and reciprocating others’ care. These challenges affected relationships of siblings, and are still present in modern societies, where it is not uncommon that full- and half-siblings, and sometimes also step-siblings, live together, or are in regular contact (see Gyuris and Kocsor, 2016).

### Rivalry and Support Among Full-Siblings

Living together with one or more siblings means that the resources available to the family have to be shared. Competition for these parental resources is basically the primary cause of sibling conflicts. Trivers (1974) theoretical framework of *parent–offspring conflicts* puts into focus the thriving of individuals to maximize their fitness by obtaining the highest possible amount of food and care from the parents. In the case of more than one offspring in the litter—speaking about humans, in the household—they also have to compete with each other. However, the intensity of competition depends on multiple factors. It is more intense when resources are scarce or when the parents distribute these unevenly among the siblings. The theories of parental investment and discriminative parental care assume that parents tend to invest more in their older, healthier children (Trivers, 1985), and it is supported by empirical data as well (Mann, 1992). The frequency of conflicts is higher between male siblings than between females (Tanskanen et al., 2016). Younger age, small age difference, and physical closeness also enhance siblings’ competition (Michalski and Euler, 2007; Barlay and Péley, 2016), a fact that is revealed by data showing that rivalry between siblings decrease after puberty, and after having moved to separate locations, physical contacts become less frequent (Pollet and Hobben, 2011). Having reached young adulthood, the amount of competition decreases, and siblings rely on each other’s support to an increasing extent (Pollet and Hobben, 2011). This behavior is influenced by marital status so that single or childless individuals provide more help to their siblings who have children (Connidis, 1992). Regarding sex differences, older sisters give the most support in the majority of cultures (Cicirelli, 1994). However, among the Ache in Amazonia, support from young males is more dominant, as with their hunting activity, they contribute to the well being of their younger siblings and parents until their marriage, typically happening around 20–21 years of age (Hurtado and Hill, 1996).

### Rivalry and Support Among Half-Siblings

In accord with the kin selection theory and the Hamilton rule (Hamilton, 1964), owing to the 50% of shared genes, on average, people are willing to support full-siblings to a larger extent than cousins with their 12.5% of shared genes, or half-siblings with their 25% (Emlen, 1997; Neyer and Lang, 2003). Support of family members depends proportionally on the genetic coefficient and on the extent to which benefits of supporting a relative exceed its costs. A study of Jankowiak and Diderich (2000) conducted in a Mormon community gives a spectacular demonstration of inclusive fitness overarching within-family relations. Mormons, practicing polygyny, live in families with children who could be either full- or paternal half-siblings. Children are expected not to discriminate between differentially related siblings. However, despite the ideological pressure in childhood, full-siblings are preferred in adulthood: people feel themselves closer to their full-siblings, they are more likely to visit family occasions such as birthdays and marriages, and provide more help both physically (e.g., in the form of childcare) and financially. In line with this, it has been shown in a cross-cultural study that older siblings show more altruism toward younger full-siblings and maternal half-siblings than paternal half-siblings (Sznycer et al., 2016). Though the average relatedness of paternal and maternal half-siblings is the same, their relationship is qualitatively different; the interactions between maternal half-siblings are more frequent than between paternal ones (Pollet, 2007; Tanskanen and Danielsbacka, 2014). The reason for this is that in Western societies, after divorce, children usually stay with the mother and grow up with children from subsequent relations of their mother (De Graaf, 1997, cited in Pollet, 2007). Co-residence makes more interactions possible, which can manifest in more care, but also in more quarreling. Besides, in contrast to maternity, paternity involves some uncertainty (Laham et al., 2005); hence, the expected genetic benefits of helping a paternal half-sibling are lower.

Similar to what was observed in full-siblings, the relationship of half-siblings is more conflict laden in early childhood than in young adulthood. In contrast, age is not a relevant factor in conflicts among step-siblings (Michalski and Euler, 2007). Highlighting the decline in the amount of conflicts with age, Tanskanen et al. (2016) showed that older (62–67 years) siblings have fewer conflicts than younger (20–40 years) siblings. In the younger age group, conflicts between full-siblings were more intense compared to half-siblings. In the same sample, maternal half-siblings had more severe conflicts than paternal half-siblings. There was also a significant difference in the intensity of debates in the older age group between full-siblings and paternal half-siblings, but not between the two types of half-siblings. These differences might be explained with the theory of parent–offspring conflicts (Trivers, 1974). One of the most important resources for survival is parental care, and siblings compete for the attention of their parents. As full-siblings consider both the mother and the father as potential caregivers, their interest is to outcompete each other to obtain more attention from them. In contrast, half-siblings have only one parent to whom they are equally related. This way, competition among them for parental attention is limited to this person, while they can approach someone else for additional provision.

### Kinship Detection

Providing more help and resources for those who are closer in genetic relatedness, and this way enhancing inclusive fitness, is only possible if there is a valid cue to rely on when assessing the degree of kinship. Mateo (2003) distinguished four different theoretical types of kin recognition mechanisms (see also Krupp et al., 2011; Kocsor, 2016). Animal studies focused mainly on the role of *phenotype matching* in kin detection (Mateo and Johnston, 2003), for which olfactory or visual cues serve as input. For example, studies with rodent species showed that related individuals adjust the amount of prosocial behavior and antagonism toward each other even if they were reared apart (Holmes and Sherman, 1982, 1983). However, s*patial cues*, such as co-residence, and *early association* with conspecifics, could also be sources of information on which kin recognition can be based (Mateo, 2003, 2015; Lieberman et al., 2007; Lieberman and Billingsley, 2016). Relying on physical and spatial cues simultaneously is a widespread heuristic in the animal kingdom to maximize inclusive fitness (Park et al., 2008). Empirical evidence suggests that, based on physical cues, humans are able to recognize kin above chance. Moreover, attributed kinship also leads to an enhanced willingness for benevolent behavior. For instance, it has been shown that maternal perinatal association increases siblings’ altruism (Lieberman et al., 2007; Sznycer et al., 2016). This finding is also supported by experiments in which facial resemblance has been manipulated (e.g., DeBruine, 2002; Platek et al., 2004, 2005), benefiting from the fact that in our species, the primary source of information is facial appearance. Besides, although humans are mainly visual, olfactory cues are also reliable when it comes to kinship detection (see Mateo, 2015).

Despite the great number of physical cues of genetic relatedness, such as facial resemblance and olfactory cues, people are not always aware of the actual degree of kinship of others around them. Though across hunter–gatherer tribes—and probably in most extinct hominid species—the stability of group constitution shows high variability (for overview, see Kelly, 2013), co-residence, and early association with others give a good approximation of the degree of relatedness. More specifically, those who live with the same individual who ensures provision (typically the mother), are likely to be more closely related (probably maternal half- or full-siblings) than those whose caretakers are different (paternal half-siblings, more distant relatives, or unrelated individuals). Hence, the fitness-relevant challenge to optimally allocate resources and reciprocate deeds differentially among kin can be answered by taking the frequency of encounters in the childhood environment into account. Once the degree of relatedness is assessed, measures to enhance inclusive fitness can be taken.

### Aims and Hypotheses

Based on the above theoretical considerations, we wished to explore whether people differentiate between siblings in their fitness-relevant decisions, such as support and reciprocation. As a first step on this road, in the current study, we focused on the retrospective assessment of experienced emotional closeness toward, and the intensity of conflicts between, siblings during childhood. Typical methods to measure cooperation and emotional closeness within the family include asking to recall memories of sharing resources or analyzing answers about hypothetical situations (Sznycer et al., 2016). However, the reliability of retrospectively assessing the support given to and received from siblings—particularly if single questions are used (e.g., Tanskanen and Danielsbacka, 2019)—is questionable. To avoid such uncertainty, in the current study, we wanted to benefit from the existence of the numerous validated questionnaires that estimate emotional closeness and conflicts experienced in childhood. The Sibling Relationship Questionnaire (SRQ) used here is a reliable tool, and we think the emotions evoked by childhood interactions are good indices for the overall quality of the relation.

Considering theories about kinship detection cues (Lieberman et al., 2007; Krupp et al., 2011; Mateo, 2015; Lieberman and Billingsley, 2016), we hypothesized that co-residence might be a proxy to assess genetic relatedness. However, because of the functioning of other kin recognition mechanisms that are based on physical cues and are more directly anchored to biological kinship, factual genetic relatedness could also have a significant effect on the quality of sibling relations. Our predictions were the following:

1. The more time one spent together with the siblings in the same family, the higher the perceived closeness toward them.
2. Relationship quality is better (e.g., more prosocial behavior) between full-siblings than half- and step-siblings. Because of paternal uncertainty, maternal full-siblings experience more warmth than paternal half-siblings even if the time spent together is controlled for.
3. Conflicts are more intense and more frequent between full-siblings than between half-siblings if the frequency of encounters is controlled for.

## Materials and Methods

### Participants

An online tool (Psytoolkit) (see Stoet, 2010, 2017) was used for data collection. After filtering out those participants who started taking the test but did not finish it, 196 people (mean age = 29.3, SD = 10.5, 38 males) between 18 and 68 remained in the sample. They differed in the number of siblings they have, ranging from 1 to 6 (mean = 1.94, SD = 1.19). However, participants were not required to complete the questions for all of their siblings, and answers to be given were limited to five siblings (mean = 1.74, SD = 1.05). Hence, we eventually had five subjects with completed questionnaires for five, 12 for four, 15 for three, 48 for two, and 116 for one sibling. This adds up to 330 completed tests. Distribution of sibling types and frequency of encounters are shown in Tables 1, 2.

**TABLE 1:** Frequencies of sibling types in the whole sample.

Sibling types	Counts	% of total	Cumulative %
Full-sibling	155	47.0	47.0
Maternal half-sibling	80	24.2	71.2
Paternal half-sibling	74	22.4	93.6
Stepsibling	21	6.4	100.0

**TABLE 2:** Distribution of frequencies of sibling encounters.

Frequency of encounters			Counts	% of total	Cumulative %
Occasionally			37	11.2	11.2

	Once in a month	17			
Monthly			41	12.4	23.6
	Several times a month	24			

	Once a week	16			
Weekly	Several times a week	21	41	11.2	36.1
	Daily	4			

Lived together			211	63.9	100.0

From the total sample, 211 answers were submitted for siblings with whom the participants lived together during their childhood. These questionnaires came from 136 subjects (mean age = 29.8, SD = 10.6, 27 males). Distribution of sibling types is shown in Table 3.

**TABLE 3:** Distribution of sibling types among those who lived together.

Levels	Counts	% of total	Cumulative %
Full-sibling	96	70.6	70.6
Maternal half-sibling	31	22.8	93.4
Paternal half-sibling	7	5.1	98.5
Stepsibling	2	1.5	100.0

### Questionnaires

Participants were asked to complete the Sibling Relationship Questionnaire (SRQ; Furman and Buhrmester, 1985; Barlay and Péley, 2016). The questionnaire consists of 27 items, arranged into 10 primary factors (see Table 4). These factors can be aggregated into three main factors. In the original test two of the factors, *maternal* and *paternal partiality* are calculated as a divergence from the “ideal state,” when participants report that their parents provided equal attention and care to both parties of the sibling pair. However, we calculated these factors to be able to assess which of the two siblings was more in the focus of the two parents. Lower scores mean that the participant felt that more attention was paid by their parents toward their sibling. In contrast, the main factor *parental partiality* was calculated as suggested by the original paper (Furman and Buhrmester, 1985; Barlay and Péley, 2016), the same way as two of the other main factors, *closeness* and *conflict* (see Table 5 for descriptives). The *communality* score aggregates all of the main factors. As the factors consist of different numbers of items, the range of scores varies widely, thus we calculated *communality* by subtracting the Z-scores of the *conflict* and *parental partiality* subscales from the *closeness* Z-score. The higher the *communality* score, the better the relationship between siblings.

**TABLE 4:** Factor structure and reliability indices of the SRQ.

Factor number	Primary factors	Main factors	Cronbach’s alpha		Aggregated factor
F1	Intimacy		0.931		
F2	Companionship		0.896		
F3	Similarity		0.829		
		Closeness/warmth		0.954	
F4	Admiration of sibling		0.917		
F5	Admiration by sibling		0.934		Communality = (Z closeness – Z conflict – Z parental partiality)
F6	Prosocial behavior		0.922		

F7	Quarreling		0.871		
		Conflict		0.854	
F8	Competition		0.777		

F9	Maternal partiality		0.906	0.882	
		Parental partiality			
F10	Paternal partiality		0.922		

**TABLE 5:** Descriptives of closeness and conflict Z-scores for different sibling encounters and sibling types.

Sibling encounters	Sibling type	Z-score closeness		Z-score conflict	
		Mean	SD	Mean	SD
Occasionally	Full sibling	0.885	NaN	–0.708	NaN
	Maternal half sibling	0.152	0.303	–0.637	0.581
	Paternal half sibling	–1.10	0.922	–0.609	0.805
	Stepsibling	–1.65	0.550	–0.0356	1.18
Monthly	Full sibling	–0.0224	0.815	1.00	0.284
	Maternal half sibling	–0.963	0.979	–0.575	0.636
	Paternal half sibling	0.160	0.984	–0.789	0.593
	Stepsibling	–0.819	1.16	–0.139	0.566
Weekly	Full sibling	0.197	0.936	0.0999	0.647
	Maternal half sibling	0.0112	0.700	–0.902	0.481
	Paternal half sibling	–0.230	1.03	–0.118	1.04
	Stepsibling	–0.0695	0.521	–0.336	0.911
Lived together	Full sibling	0.205	0.907	0.460	1.01
	Maternal half sibling	0.111	0.992	–0.159	0.921
	Paternal half sibling	0.412	0.907	0.0881	0.989
	Stepsibling	–0.258	0.673	0.154	0.334

### Analysis 1—Effects of Frequency of Encounters and Genetic Relatedness on Sibling Relations

#### Determination of Best-Fitting Models

We ran a series of Generalized Linear Mixed Models (GLMM; Laird and Ware, 1982) in SPSS 25.0 to assess which of the potential factors contribute the most to the quality of sibling relations (Table 6). We used factors of the SRQ as target variables with normal probability distribution and identity as a link function. As predictors, we set *relative age difference* between siblings, *sex of sibling pairs* (i.e., both females, both males, or mixed), *relatedness*, *frequency of encounters*, and the interaction between relatedness and frequency of encounters. As noted in the section “Participants,” subjects varied in the number of siblings and also in the number of siblings they completed the questionnaires for. Because of that, participants completed a varying number of SRQ tests from one to five, each referring to a different sibling. Therefore, in each analysis, *siblings’ ID* was used as the level of repeated measures and *participants’ ID* as a random variable with intercept included. For *post hoc* pairwise comparison, sequential Bonferroni was used. Our strategy was to create models with all possible variables and interactions of interest, then to omit those variables from the models that were the least significant, one after the other. The iterations continued until a significant model with the highest possible number of significant predictors could be determined.

**TABLE 6:** *p*-Values of fixed effects of variables and interactions included in the final GLM models to estimate predictors of Sibling Relationship Questionnaire (SRQ) scores.

Target variable		Akaike corrected IC	Fixed effects (*p*-values)^a^					
			Corrected model	Sex of sibling pair	Sibling type	Frequency of encounters	Sibling type × Frequency of encounters	Age difference
F1	Intimacy	1009.962	<0.001	<0.001	<0.001	–	<0.001	
F2	Companionship	938.626	<0.001		0.001	<0.001		
F3	Similarity	845.372	<0.001		0.007	–	<0.001	
F4	Admiration of sibling	968.905	<0.001		<0.001	–	<0.001	
F5	Admiration by sibling	975.876	<0.001		<0.001	0.004	0.004	0.020
F6	Prosocial behavior	968.923	<0.001	0.004	<0.001	–	<0.001	
F7	Quarreling	948.620	<0.001		0.001	0.005		0.049
F8	Competition	882.221	<0.001		<0.001	–		0.003
F9	Maternal partiality	739.663	<0.001		<0.001	–	0.001	
F10	Paternal partiality	757.016	<0.001		0.001	–	<0.001	
Closeness		1839.532	<0.001		<0.001	0.019	0.008	
Conflict		1224.985	<0.001		<0.001	–		0.001
Partiality		929.520	<0.001		0.003	<0.001		
Communality		1218.811	<0.001			–	<0.001	0.029

*^*a*^Blank fields indicate that the factor was not included in the final model. All factors in the models were significant on a *p* < 0.05 level.*

#### Results

The best-fitting GLM models show that the effects of frequency of encounters and relatedness are in line with the predictions. First, higher frequency of encounters had a significant positive effect on the scores of *companionship* and *admiration by sibling* factors, and on the main factor *closeness* as well (Table 6) (see also the project’s OSF site via http://bit.ly/halfsib_output for detailed statistics of fixed effects and coefficients). Moreover, we found a significant positive effect on *quarreling* and a negative effect on *parental partiality*, suggesting that those who meet only occasionally are less likely to engage in debates, and also feel they are treated better by their parents than their siblings do.

The second variable in focus, that is relatedness, had a significant positive effect on each of the primary and the main factors, but no effect on the aggregated *communality* factor. Hence, a higher degree of genetic relatedness enhanced the feeling of closeness and warmth, but also predicted more conflicts between full-siblings than between half-siblings. *Maternal partiality* scores were higher for paternal, whereas *paternal partiality* scores for maternal half-siblings. To put it another way, siblings seem to have experienced some kind of bias from the parents toward their biological children.

When the relative difference in age was higher, scores on the *admiration by sibling* and *communality* factors increased, whereas scores on *quarreling*, *competition*, and therefore also *conflicts*, decreased. *Intimacy* scores of sisters were significantly higher compared to brothers or mixed-sex sibling pairs, and *prosociality* scores increased significantly with each female in a sibling pair.

The interactions in the GLM models, however, show that the connection between the frequency of encounters and relatedness is more complicated than expected. We received significant interactions (see Table 6) in seven of the primary factors (*intimacy*, *similarity*, *admiration of sibling*, *admiration by sibling*, *prosocial behavior*, *maternal*, and *paternal partiality*), and the main factor *closeness*. The model for *communality* score also includes the interaction as a significant factor. All of these interactions suggest that overall relationship quality did not differ between siblings with different levels of relatedness when they lived together or met at least once a week. The significant main effects of relatedness were caused by those sibling pairs who saw each other only monthly or even more rarely.

#### Controlling for Potential Bias in Questionnaire Completion

As noted in the section “Participants,” the number of participants’ siblings ranged from 1 to 6; however, the number of siblings for which they could complete the questionnaires was limited to 5. Besides, they were not required to complete the task for all siblings. This put a potential bias in the data, as participants might have refused to answer questions about their siblings with whom their relationship was problematic. Therefore, we compared the SRQ factor scores of those who completed the questionnaires for all of their siblings versus those who did this for only a subset of siblings. Because the distribution of the factor scores were not normal (Shapiro–Wilk test), we used Welch’s independent sample *t*-test in Jamovi 1.1.9.0. None of the differences were significant (Table 7). Hence, the statistical results suggest that the findings are very likely not the consequence of a biased completion of the tests.

**TABLE 7:** Statistical results of the test of normality (Shapiro–Wilk) and the independent samples *t*-tests (Welch’s *t*) comparing those who completed the questionnaires for all siblings versus those who did not.

Factor	Shapiro–Wilk W*	Welch’s *t*-test			
		*t*	df	*p*	Cohen’s *d*
F1—Intimacy	0.921	0.020	66.952	0.984	0.003
F2—Companionship	0.958	0.359	67.233	0.721	0.056
F3—Similarity	0.971	–1.834	75.982	0.071	–0.252
F4—Admiration of sibling	0.896	–0.508	71.225	0.613	–0.074
F5—Admiration by sibling	0.921	–0.529	68.582	0.598	–0.080
F6—Prosocial behavior	0.935	–0.875	71.945	0.384	–0.126
F7—Quarreling	0.939	0.293	65.757	0.771	0.047
F8—Competition	0.839	1.555	66.689	0.125	0.245
F9—Maternal partiality	0.914	–0.152	59.214	0.880	–0.025
F10—Paternal partiality	0.887	–0.002	68.417	0.999	0.000
Closeness	0.958	–0.777	74.256	0.440	–0.109
Conflict	0.939	1.007	66.037	0.318	0.161
Parental partiality	0.883	0.924	74.729	0.359	0.118
Communality	0.971	–1.387	64.413	0.170	–0.203

*^∗^All *p* < 0.001.*

### Analysis 2—Effect of Relatedness on the Relation Types of Co-resident Siblings

#### Determination of Relation Types of Co-resident Siblings

**FIGURE 1:**
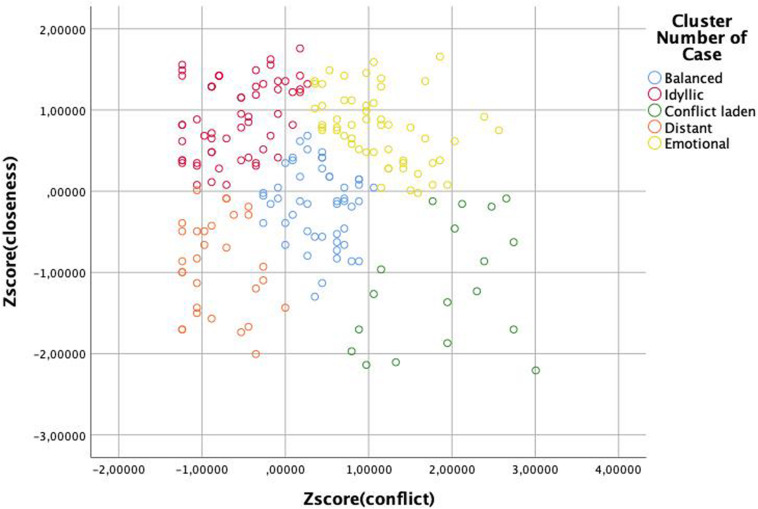
Clusters of relation types of siblings as determined by the Z-scores of the closeness and conflict factors.

As the first analysis of potential predictors of sibling relations showed that there is an interaction between genetic relatedness and frequency of encounters, we decided to scrutinize the relation of co-resident siblings further. Keeping in mind that due to the overlapping sample this analysis is not fully independent of the previous one, we wished to explore whether closer genetic relatedness is associated with better relationship quality of co-resident siblings. In their study, Barlay and Péley (2016) reported that full-siblings can be grouped into five categories according to their closeness and conflict scores: *idyllic*, *emotional*, *conflict laden*, *distant*, and *balanced*. To test whether there is a difference between the relation type of different sibling types as well, first, we had to determine whether sibling pairs in our sample can be grouped into these same categories. Following the rationale of that study, we ran a K-mean cluster analysis on the Z-scores of *closeness* and *conflict* sub-scales to group the sub-sample of co-resident siblings into five clusters. The results of the ANOVA analyses on the mean scores of the sub-scales of the groups (Figure 1, Tables 8–10) demonstrate that *relation types* correspond to the pattern observed in the previous study (Barlay and Péley, 2016). Though using relation type categories instead of continuous factor scores necessarily leads to some simplification of the data, it also makes the interpretation of the results easier. Therefore, we used these variables in the next analysis.

**TABLE 8:** Welch’s one-way ANOVA testing the differences of the scores of closeness and conflicts between relation type categories.

SRQ factor	*F*	df1	df2	*p*
Z-score closeness	99.5	4	71.5	<0.001
Z-score conflict	161.6	4	73.4	<0.001

**TABLE 9:** Games–Howell *post hoc* test for testing differences of the closeness Z-scores between relation type categories.

Relation type		Idyllic	Conflict laden	Distant	Emotional
Balanced	Mean difference	−1.08	1.01	0.750	−0.947
	*t*-Value	−11.9	5.26	5.97	−10.54
	df	102	21.9	51.7	98.4
	*p*-Value	**<0.001**	**<0.001**	**<0.001**	**<0.001**
Idyllic	Mean difference		2.08	1.826	0.129
	*t*-Value		11.00	14.89	1.50
	df		21.1	48.6	110.0
	*p*-Value		**<0.001**	**<0.001**	0.563
Conflict laden	Mean difference			−0.256	−1.954
	*t*-Value			−1.23	−10.34
	df			29.0	20.9
	*p*-Value			0.736	**<0.001**
Distant	Mean difference				−1.698
	*t*-Value				−13.91
	df				47.7
	*p*-Value				**<0.001**

*Figures in bold indicate that the difference is significant on a *p* < 0.05 level.*

**TABLE 10:** Games–Howell *post hoc* test for testing differences in the conflict Z-scores between relation type categories.

Relation type		Idyllic	Conflict laden	Distant	Emotional
Balanced	Mean difference	0.989	−1.50	1.222	−0.719
	*t*-Value	12.6	−8.54	14.57	−8.16
	df	104	20.1	59.0	92.3
	*p*-Value	**<0.001**	**<0.001**	**<0.001**	**<0.001**
Idyllic	Mean difference		−2.49	0.232	−1.708
	*t*-Value		−13.89	2.57	−18.07
	df		21.6	71.3	105.1
	*p*-Value		**<0.001**	0.088	**<0.001**
Conflict laden	Mean difference			2.725	0.784
	*t*-Value			14.99	4.27
	df			22.5	23.6
	*p*-Value			**<0.001**	**0.002**
Distant	Mean difference				−1.941
	*t*-Value				−19.61
	df				78.0
	*p*-Value				**<0.001**

*Figures in bold indicate that the difference is significant on a *p* < 0.05 level.*

#### Determination of Best-Fitting Models

For analyzing the relations of co-resident siblings, we followed the same strategy as for the analysis of the whole sample. Similarly, we ran a series of GLMMs to find the best predictors that determine sibling relations. As the number of co-resident step-siblings and paternal half-siblings in the sample was very limited (Table 3), only full-siblings and maternal half-siblings were included. In contrast to the first analysis where target variables were primary factors and main factors of relation quality, we used the categorical variable *relation type* as a target variable with multinomial probability distribution and generalized logit link function. *Participants’ ID* was used as a random variable with intercept included. Adjustments for multiple comparisons were made with sequential Bonferroni correction. We started with predictors *relative age difference* between siblings, *sex of sibling pairs*, and *relatedness*, then omitted the least significant variables in each iteration until a significant model with the highest possible number of significant factors was obtained. Statistical parameters of the best fitting models are shown in Tables 11, 12.

**TABLE 11:** Statistical values of the fixed effects of the best-fitting GLM models to estimate predictors of siblings’ relation type categories.

Reference category	Akaike Corrected IC		Fixed effects			
			*F*	df1	df2	*p*
Balanced	2732.945	Corrected model (Age difference)^a^	4.131	4	182	**0.003**
Idyllic	2766.639	Corrected model (age difference)	4.055	4	182	**0.004**
Conflict laden	2732.945	Corrected model (age difference)	4.131	4	182	**0.003**
Distant	2752.108	Corrected model (age difference)	4.251	4	182	**0.003**
Emotional	2749.833	Corrected model (age difference)	4.117	4	182	**0.003**

*^*a*^The only variable in the model is age difference; therefore, fixed effects are the same for the corrected model. Figures in bold indicate that the effect is significant on a *p* < 0.05 level.*

**TABLE 12:** Statistical values of the fixed coefficients of the best-fitting GLM models to estimate predictors of siblings’ relation type categories.

Reference category	Target category	Fixed coefficients			
		Intercept		Age difference	
		*p*	Exponential coefficients	*p*	Exponential coefficients
Balanced	Idyllic	0.830	0.500	**0.023**	**1.156**
	Conflict laden	0.925	0.735	0.210	0.880
	Distant	0.677	0.259	**0.029**	**1.163**
	Emotional	0.905	1.472	0.437	0.948
Idyllic	Balanced	0.859	1.798	**0.030**	**0.878**
	Conflict laden	0.933	1.323	**0.010**	**0.772**
	Distant	0.818	0.467	0.743	1.020
	Emotional	0.766	2.668	**0.004**	**0.831**
Conflict laden	Balanced	0.912	1.360	0.210	1.136
	Idyllic	0.890	0.680	**0.008**	**1.314**
	Distant	0.709	0.353	**0.009**	**1.322**
	Emotional	0.802	2.002	0.469	1.077
Distant	Balanced	0.651	3.839	**0.024**	**0.858**
	Idyllic	0.829	1.903	0.918	0.994
	Conflict laden	0.729	2.820	**0.008**	**0.755**
	Emotional	0.561	5.644	**0.004**	**0.814**
Emotional	Balanced	0.910	0.689	0.483	1.048
	Idyllic	0.747	0.344	**0.003**	**1.212**
	Conflict laden	0.838	0.507	0.421	0.922
	Distant	0.602	0.178	**0.005**	**1.219**

*Figures in bold indicate that the coefficient is significant on a *p* < 0.05 level.*

#### Results

*Relative age difference* was the only variable significantly associated with siblings’ belonging into one of the relation type categories. The general pattern is that with the increase in age difference, the likelihood of getting into a category with fewer conflicts and more warmth is increasing as well. For instance, if the age difference between two siblings is increased by 1 year, the chance that siblings’ relationship type is idyllic (with high closeness and low conflict scores) or distant (with low closeness and conflict scores), rather than balanced (with closeness and conflict scores around the mean) is about 16% higher (Table 12).

## Discussion

In this study, we tested the evolutionary-inspired assumption that sibling relations conform to the enhancement of inclusive fitness—i.e., the increase of genetic representation in the next generation—and are influenced by the possibility of reciprocation and by the competition for resources available in the family. In advance, as a note of caution, we would like to point out that our sample was limited in both size and cultural diversity. Therefore, referring to the results as universally valid would be an exaggeration (Henrich et al., 2010). However, we may draw some conclusions that could be the subjects of future cross-cultural verification. According to our first hypothesis, the time spent together has a positive influence on the feeling of closeness. This has been confirmed by the GLMM’s showing that the frequency of encounters has a significant effect on sibling relations in childhood, both through increasing the scores on factors related to cooperation and emotional closeness, and decreasing the intensity and amount of conflicts.

The second hypothesis was related to the effect of relatedness, whereby we assumed the manifestation of *Hamilton’s rule* (Hamilton, 1964). We expected that with the increase in the genetic coefficient, the feeling of closeness—approximated by the memories of childhood emotions toward siblings—will also rise. Yet, previous studies (Tanskanen et al., 2016) highlighted that competition could be more intense between full-siblings in contrast to half-siblings because both of them depend on the investment of the two co-resident parents to an equal extent. Since half-siblings can demand—and receive—more care and support from one of the parents (who does or does not live in the same household), the competition for parental investment will be diluted. This is due to the asymmetric pattern of genetic relatedness within the functional family, as in patchwork families typically there is a parent with whom not all of the children share genes. The current results support these earlier findings. In addition, prosocial behavior was significantly higher between maternal half-siblings than between paternal ones.

However, the latter effect was mainly driven by the data of those who met only occasionally. This is revealed by the perhaps most interesting result of the current study, namely, the interaction between frequency of encounters and genetic relatedness. To sum up, the statistical analyses indicate that there is no difference between step-, half-, and full-siblings in terms of relationship quality if they lived together or met frequently. In contrast, the relation of siblings who lived apart and met rarely follow the pattern predicted by the evolutionary theories of inclusive fitness (Hamilton, 1964) and parent–offspring conflict (Trivers, 1972). Though we did not predict such an outcome, it echoes the recent findings of Tanskanen and Danielsbacka (2019) who analyzed a large sample from German birth cohorts. They found that adults have more contacts and feel themselves emotionally closer to full-siblings if they were raised separately, but there is no difference between the two sibling types if they lived together in childhood. The results may not be surprising if the concept of fitness interdependence, focusing on kinship terminologies (Cronk et al., 2019), is taken into account. Having scrutinized patterns of kin terms across cultures, Cronk et al. (2019) concluded that in most cultures, these terms map only superficially onto patterns of real genetic relatedness. These terms refer more to the interdependence of individuals within a group with respect to the enhancement of each other’s fitness, and by doing so, tightens the fabric of the social network. Rearing children together may also evoke a feeling of kinship that is not so much related to biological facts but to the act of nurturance (Holland, 2012). It is also possible that parents take advantage of using those terms, which suit their fitness needs the best, and by emphasizing children’s being siblings and obscuring the information of being only half- or step-siblings, they try to promote affection. This possibility emerges particularly when the siblings live together. Besides, the concept of fitness interdependence suggests that even if the individuals reared together are not genetically related, their mutual dependence on the same caregivers creates circumstances under which their genetic interests overlap and their future reproductive success can be enhanced by supportive behavior toward each other (Cronk et al., 2019).

In addition, frequent encounters enhance the possibility to reciprocate favors, which in turn promotes cooperation. This has also been highlighted by studies, which concluded that longer co-residence duration of siblings is associated with more altruistic behavior (Anderson et al., 1999a, b; Sznycer et al., 2016; Tanskanen and Danielsbacka, 2019). In contrast, when events of common acts occur sparsely, there is less chance to reciprocate others’ good deeds. However, it is a remarkable result of our study that relation quality matched the pattern predicted by Hamilton’s rule in those sibling pairs who met only occasionally. Paternal uncertainty (Buss and Barnes, 1986; Greiling and Buss, 2000) might well account for the better relation between maternal half-siblings, albeit the mediating role of mothers, who play a different role within the family, cannot—and should not—be excluded (keeping in mind that the second explanation is not fully independent of the first one). Mothers may, presumably, catalyze and strengthen siblings’ cooperation. The exact way of how this happens requires additional research.

The closer analysis of co-resident siblings’ relation type—echoing the preceding analysis—did not show any connection between the degree of relatedness and relationship quality either. In contrast to the third hypothesis, Hamilton’s rule could not be caught by the SRQ scores. Nevertheless, the proportional improvement of relationship quality with the increase in age difference is consonant with previous results (Pollet and Hobben, 2011).

Though we did not test both cue-based and rule-based kin-recognition mechanisms (Park et al., 2008) directly, it is probable that both serve as input for enhancing inclusive fitness. In this study, we found that in patchwork families, rule-based mechanisms have a greater impact on siblings’ relations. Early association makes genetic relatedness likely, and although sometimes this heuristic fails, the fitness interdependence of children raised together prevents it from being selected against. It is also important to note that parents might have a catalyzing role in improving sibling relations. Along with a systematic analysis of this role, future studies should also address long-term effects of rearing children with diverse genetic relatedness together, such as their relationship quality and willingness to cooperate in adulthood.

## Data Availability Statement

The datasets for this study can be found in the Open Science Framework repository (http://bit.ly/halfsib_output).

## Ethics Statement

The United Ethical Review Committee for Research in Psychology, Hungary, reviewed and approved the project proposal. The participants provided their written informed consent to participate in this study.

## Author Contributions

PG and FK conceived the study design and wrote the draft of the manuscript. FK, AL, and LK discussed the statistical analyses. FK carried out the analyses. ZsK, FK, and TF contributed to data collection and data processing. AL and LK helped prepare the final version of the manuscript. LK did the proof-reading, including grammar- and spell-checking.

## Conflict of Interest

The authors declare that the research was conducted in the absence of any commercial or financial relationships that could be construed as a potential conflict of interest.
